# Risk and factors related to the development of lesions due to xerosis in hospitalized elderly people

**DOI:** 10.17533/udea.iee.v43n1e03

**Published:** 2025-04-14

**Authors:** Ronny Anderson de Oliveira Cruz, Carla Braz Evangelista, Mirian Alves da Silva, Cleide Rejane Damaso de Araújo, Jacira dos Santos Oliveira, Marta Miriam Lopes Costa

**Affiliations:** 1 Nurse, Master. Assistant Professor. Email: ronnyufpb@gmail.com. Corresponding author. https://orcid.org/0000-0001-6443-7779 Universidade Federal da Paraíba Brazil ronnyufpb@gmail.com; 2 Nurse, Ph.D. Assistant Professor. Email: carlabrazevangelista@gmail.com https://orcid.org/0000-0001-7063-1439 Centro Universitário de João Pessoa Brazil carlabrazevangelista@gmail.com; 3 Nurse, Ph.D. Adjunct Professor. Email: miads.enf@gmail.com https://orcid.org/0000-0003-2959-4642 Universidade Federal da Paraíba Brazil miads.enf@gmail.com; 4 Nurse, Ph.D. Adjunct Professor. Email: cleidedamaso@gmail.com https://orcid.org/0000-0001-7383-9737 Universidade Federal da Paraíba Brazil cleidedamaso@gmail.com; 5 Nurse, Ph.D. Adjunct Professor. Email: jacirasantosoliveira@gmail.com https://orcid.org/0000-0002-3863-3917 Universidade Federal da Paraíba Brazil jacirasantosoliveira@gmail.com; 6 Nurse. Ph.D. Adjunct Professor. Email: marthamiryam@hotmail.com https://orcid.org/0000-0002-2119-3935 Universidade Federal da Paraíba Brazil marthamiryam@hotmail.com; 7 School of Nursing, Federal University of Paraíba, João Pessoa, Paraíba, Brazil. Universidade Federal da Paraíba School of Nursing Federal University of Paraíba João Pessoa Paraíba Brazil; 8 School of Nursing, Center University of João Pessoa. João Pessoa, Paraíba, Brazil. Centro Universitário de João Pessoa School of Nursing Center University of João Pessoa João Pessoa Paraíba Brazil

**Keywords:** nursing, skin aging, elderly health, enfermería, envejecimiento de la piel, salud de las personas mayores., enfermagem, envelhecimento da pele, saúde do idoso.

## Abstract

**Objective.:**

To analyze the risk and factors related to the development of skin lesions due to xerosis in hospitalized elderly people.

**Methods.:**

This was a descriptive and cross-sectional study with 455 elderly people hospitalized in Paraíba (Brazil). The Risk Assessment Scale for the Development of Lesions Associated with Xerosis Cutis in Elderly People (ERLAX-53) developed in Brazil by Cruz in 2023 was used.

**Results.:**

The sample consisted of 272 (59.8%) elderly people from the medical clinic and 183 (40.2%) from the ICU, most of whom were females (54.5%). There were high frequencies of the variables “immobility” (58.9%), “friction and shear” (87.7%), “presence of comorbidities” (99.3%) and “predominance of dry skin” (79.8%). The correlation was positive and moderate for “mobility”, “level of consciousness”, “tactile sensitivity”, “temperature” and “phototype”. As for the risk of lesions associated with xerosis, 337 (74.1%) were at medium risk and 101 (22.2%) at high risk.

**Conclusion.:**

96.3% of the elderly people with xerosis cutis who participate in the study were at medium to high risk of developing additional skin lesions, which is why nursing must implement preventive and treatment strategies to care for these people.

## Resumen

**Objetivo.:**

Analizar el riesgo y los factores relacionados con el desarrollo de lesiones de xerosis cutánea en personas mayores hospitalizados.

**Métodos.:**

Se trata de un estudio descriptivo transversal realizado con 455 ancianos hospitalizados en Paraíba (Brasil). Se utilizó la Escala de Evaluación de Riesgo para el Desarrollo de Lesiones Asociadas a Xerosis Cutánea en Personas Mayores (ERLAX-53) desarrollada en Brasil por Cruz en 2023.

**Resultados.:**

La muestra estuvo compuesta por 272 (59.8%) ancianos de la clínica médica y 183 (40.2%) de la UCI, siendo la mayoría del sexo femenino (54.5%). Hubo altas frecuencias en las variables inmovilidad (58.9%), fricción y cizallamiento (87.7%), y presencia de comorbilidades (99.3%), y predominio de piel seca (79.8%). La correlación fue positiva y moderada en movilidad, nivel de conciencia, sensibilidad táctil, temperatura y fototipo. En cuanto al riesgo de lesiones asociadas a la xerosis, 337 (74.1%) presentaron riesgo medio y 101 (22.2%) riesgo alto.

**Conclusión.:**

El 96.3% de las personas mayores con xerosis cutánea que participaron en el estudio tuvieron entre riesgo medio y alto de desarrollar lesiones adicionales en la piel, por lo que Enfermería debe implementar las estrategias preventivas y de tratamiento para el cuidado de personas.

## Resumo

**Objetivo.:**

Analisar o risco e os fatores relacionados ao desenvolvimento de lesões por xerose cutânea em idosos hospitalizados.

**Métodos.:**

Trata-se de um estudo descritivo transversal realizado com 455 idosos internados na Paraíba (Brasil). Foi utilizada a Escala de Avaliação de Risco para o Desenvolvimento de Lesões Associadas à Xerose Cutânea em Idosos (ERLAX-53) desenvolvida no Brasil por Cruz em 2023.

**Resultados.:**

A amostra foi composta por 272 (59,8%) idosos da clínica médica e 183 (40.2%) da UTI, sendo a maioria do sexo feminino (54.5%). Houve altas frequências nas variáveis ​​“imobilidade” (58.9%), “fricção” e cisalhamento” (87.7%), “presença de comorbidades” (99,3%) e “predomínio de pele seca” (79,8%). A correlação foi positiva e moderada em mobilidade, nível de consciência, sensibilidade tátil, temperatura e fototipo. Quanto ao risco de lesões associadas à xerose, 337 (74.1%) apresentaram médio risco e 101 (22,2%) alto risco.

**Conclusão.:**

Em 96.3% dos idosos com xerose cutânea que participaram do estudo, apresentaram entre médio e alto risco de desenvolver lesões cutâneas adicionais, portanto a Enfermagem deve implementar estratégias preventivas e de tratamento para o cuidado destas pessoas.

## Introduction

Xerosis cutis is an inherent alteration in the skin that can occur at any stage of life and is related to various factors, such as inflammatory processes, unfavorable environmental conditions and chronic diseases like diabetes and kidney failure. Nonetheless, it is an inherent feature in the aging process, where the Stratum Corneum (SC) is unable to adequately maintain a water concentration gradient between the skin surface and the epidermal cells, which leads to reduced cell renewal and increased transepidermal water loss.^1^ The world’s population is aging rapidly, which is why estimates suggest that the proportion of elderly people aged 60 and over will more than double between 2015 and 2050, and there will be around 1.5 billion people aged 65 and over by 2050. Aging occurs as a result of a combination of intrinsic and extrinsic factors. Concerning the skin, the greatest impairment occurs due to the reduced water content, which, in addition to decreasing elasticity, alters the protective barrier function of this organ.^2^

The physiological alterations that occur in the skin system of elderly people are characterized by a reduction in dermal thickness, loss of elastic fibers, decrease in subcutaneous adipose tissue, reduction in capillaries in the skin and dryness. The reduction in Natural Moisturizing Factors (NHF), which act to control and maintain the natural hydration unveiled by the skin, and the impairment of the lipid layer mean that there is a greater loss of water in the Stratum Corneum (SC).^3^ Thus, the clinical signs of xerosis are dryness characterized by flaking, cracking and even inflammation, especially because it is a condition that can be accompanied by pruritus, which will, consequently, increase the risk of secondary infections. As a result of the failure to maintain an adequate water concentration gradient between the living cells of the epidermis and the surface of the skin, there is also a decrease in the production of sebum and sweat.^4^

The adverse outcomes and impacts of skin aging are becoming a global challenge for the health of elderly people, as dermal alterations have significant and widespread implications. After the age of 50, the frequency of conditions that alter the functions and appearance of the skin increases in parallel with epidermal dysfunction. Epidermal dysfunction predisposes to xerosis, pruritus, atopic dermatitis and contact dermatitis. These alterations affect up to 70% of elderly people, with xerosis and pruritus being the most common skin disorders.^5^ A study conducted with 314 elderly people living in long-term facilities in Berlin found a high prevalence of xerosis of up to 95.9%. A multicenter study with 11,602 elderly people admitted to hospitals and asylums revealed a prevalence of 34.4%, mainly located in the upper and lower limbs, with significant skin dryness. Among patients with skin care dependency, the proportion of those with dry skin was 84.7%.^2^^,^^6^ Both in research and in care practice, the various triggers of xerotic skin, the conditions considered to be at risk and the set of interventions and products used as preventive practices have been perceived. Skin care is a fundamental aspect of clinical nursing practice. In this context, it is worth highlighting issues related to hygiene and the use of products with moisturizing properties applied to the skin, as they represent care measures that contribute substantially to prevention and treatment.^7^

The attention and care given to people at risk or who already have skin lesions is primarily carried out by nurses. In order for this to be effective, a dynamic and comprehensive process with interprofessional participation is necessary. Nonetheless, both the prevention and treatment of skin lesions entail significant costs for health services and other institutions, as they require a large amount of material inputs and human resources, as well as increasing the workload. Audits carried out in public health services have revealed that inconsistencies in the management of lesions and the use of outdated methods contribute to high costs and ineffective results.^8^ Thus, one can perceive the importance of a careful assessment of the skin, which will serve as a basis for clinical reasoning and decision making, as well as the use of instruments that can alert to the risk of skin lesions, such as the Risk Assessment Scale for the Development of Lesions Associated with Xerosis Cutis in Elderly People (ERLAX-53),^9^ designed to help nurses to identify the risk score for lesions associated with xerosis and contribute to the planning and quality of care provided to this clientele. 

Skin lesion prevention protocols have demonstrated a reduction in the incidence of these problems. When integrating these protocols, risk prediction scales are often studied and implemented. Scientific production on xerosis cutis in the elderly population is diffident, and sometimes the emphasis is restricted to aspects related to dry feet due to complications of diabetes or as a risk factor for skin tears, which justifies broadening the scope of knowledge about the real conditions of the population under study, as well as corroborating the strengthening of nursing care practice through a comprehensive instrument, designed and based on the theoretical foundation of a prescriptive theory. Its guiding question is: What is the risk and its correlated factors for the development of lesions due to xerosis cutis in hospitalized elderly people assessed using the ERLAX-53? In view of the above, this study seeks to analyze the risk and factors correlated with the development of lesions due to xerosis cutis in hospitalized elderly people assessed using the ERLAX-53.

## Methods

This is a cross-sectional study carried out in a medium-sized general hospital in the metropolitan region of João Pessoa, located in the state of Paraíba, Brazil. The institution was chosen because it routinely receives a considerable number of elderly people, as well as serving as a teaching and training institution for undergraduate and residency students in hospital health. It has 50 beds in the Medical Clinic (MC) and eight in the Intensive Care Unit (ICU). The population was composed and estimated based on the number of elderly people admitted in the year prior to collection, with 706 in the clinic and 338 in the ICU. Considering a sampling error of 5% and a confidence level of 95%, the minimum sample considered sufficient was 250 elderly people admitted to the MC and 180 to the ICU. Accordingly, the non-probabilistic and convenience sample consisted of 272 and 183 respectively from each unit, making a total of 455 participants who met the inclusion criteria and agreed to participate in the research.

The inclusion criteria were: being aged 60 or over and hospitalized for at least 24 hours in the institution. For those with cognitive impairment, assessed on the basis of the mental state item on the Morse Scale, the family members/guardians were consulted as to whether or not they would authorize their participation in the study. Furthermore, those who were hemodynamically unstable or developed restrictions that made assessment impossible were excluded, as well as patients readmitted during the data collection period, which occurred between January and October 2023.

In order to collect the data, the ERLAX-53 was used in conjunction with the participants' sociodemographic data questionnaire. The scale showed evidence of validity with a Content Validity Coefficient (CVC) of 0.926, while the reliability with Cronbach's Alpha and McDonald's Omega were 0.815 and 0.942, respectively,^9^ proving to be useful for conducting preventive measures during care management. It has 15 variables and was designed from a scoping review in which the conditions that increase the risk of lesions due to xerosis in the elderly population^8^ were mapped and theoretically based on the Basic Human Needs Theory. Risk stratification is defined as low risk (15 to 27), medium risk (28 to 40) and high risk (41 to 53). The application time was approximately 10 minutes for each patient in both the MC and ICU settings and was carried out by two nurses, one of whom was a specialist nurse in dermatological nursing, together with the researcher responsible for the morning and afternoon shifts. 

In the ICU, it always occurred in the afternoon period, due to the routine visiting hours and the need for consent from those responsible. Everyone was informed about the objectives of this study and signed a consent form to participate in it. The water temperature was checked using a thermometer specifically designed for this purpose and based on kerosene. Tactile sensitivity was assessed using a Semmes-Weinstein monofilament, also known as a foot esthesiometer. In patients with bilateral amputation, the test was carried out on the hands according to the recommendations for applying the scale. 

It was decided to analyze which variables presented a correlation with high risk. To this end, the Spearman’s correlation test was used, analyzing ranking positions, where the closer to -1 or + 1, the stronger the correlation. Positivity indicates that the variables are directly proportional, while negativity is inversely proportional. A value of zero indicates that there is no correlation. The parameters for interpreting the size of the effect are displayed in Table 1.^10^

**Table 1:** Values and meaning for correlation analysis. João Pessoa, PB, Brazil 2023

Value	Meaning
-1	Strong and perfect negative correlation
-0.9 a -0.99	Very high negative correlation
-0.7 a -0.89	High negative correlation
-0.4 a -0.69	Moderate negative correlation
-0,2 a -0,39	Low negative correlation
-0.01 a -0.19	Very low negative correlation
0	Null correlation
0.01 a 0.19	Very low positive correlation
0.2 a 0.39	Low positive correlation
0.4 a 0.69	Moderate positive correlation
0.7 a 0.89	High positive correlation
0.9 a 0.99	Very high positive correlation
1	Large and perfect positive correlation

The significance level used was *p*<0.001. After the data was collected, it was entered into the Excel for Windows program and then transferred to the Statistical Package for the Social Sciences (SPSS) software, version 27.0, where descriptive statistical analyses of distribution and frequency were carried out, as well as Spearman’s correlation test between variables and high risk. 

The research was conducted in accordance with Resolution 466/2012 of the Brazilian National Health Council, as well as Resolution 564/17 of the Brazilian Federal Nursing Council, obtaining approval under CAAE nº 60658022.8.0000.5188 and Opinion nº 5.626.694. All participants were informed of the objectives of this study and signed a consent form to participate in it.

## Results

Table 2 presents the description of the sociodemographic characterization of the 455 elderly people to whom the ERLAX-53 was applied. It should be underlined that most of them were hospitalized in the MC were females, lived with family members and were retired. The average age was 78.5 years (SD = 10.7; minimum = 60; maximum = 97). Of these, 170 (37.4%) were married and 305 (67%) had a family income of two minimum wages or more. 

**Table 2:** Sociodemographic variables of the 455 hospitalized elderly people. João Pessoa, PB, Brazil, 2023

Variables	***n* (%)**
Unit	
Medical clinic	272 (59.8%)
ICU	183 (40.2%)
Gender	
Male	207 (45.5%)
Female	248 (54.5%)
Marital status	
Married	170 (37.4%)
Single	108 (23.7%)
Widowed	170 (37.4%)
Stable union	07 (1.5%)
Family arrangement	
Family	388 (85.3%)
Alone	54 (11.9%)
LTCF^*^	13 (2.9%)
Occupation	
Retired	332 (73%)
INSS Benefit**	45 (9.9%)
Self-employed	78 (17.1%)
Family income	
Up to a minimum wage	150 (33%)
From two minimum wages	305 (67%)

* Long-Term Care Facility; **Brazilian National Institute of Social Security; Value of the minimum wage in the collection period: R$ 1320/ US$ 241.

The results of the 15 variables assessed during the application of the ERLAX-53 are displayed in Table 3, where it can be observed that, among the highest frequencies, immobility or very limited stand out, which together were present in 303 elderly people (66.6%). Friction and shear were found in 399 (87.7%), presence of comorbidities in 399 (87.7%) and decreased turgor and elasticity in 452 (99.3%), in addition to absence of tactile sensitivity in 199 (43.7%). 

Of the total sample, 268 (58.9%) were intensively sedated or comatose, 363 (79.8%) had dry skin texture and 169 (37.1%). associated with deep wrinkles (37.1). Pruritus was absent in 441 (96.9%) of the sample, 206 (45.35%) of whom used any available hydrating product (also known as moisturizer) and 232 (51%) of whom said they applied it once a day. The water temperature for bathing was below 34° in 301 (66.2%) and 182 (40%) were eutrophic. White skin was the most common phototype in 142 (31.2%). Furthermore, 337 (74.1%) were at medium risk of developing lesions and 101 (22.2%) were at high risk. 

**Table 3:** Distribution of the results of the application of the ERLAX-53. João Pessoa, PB, Brazil 2023

Variables	***n* (%)**
Age	
60-69	210 (46.2%)
70-79	111 (24.4%)
>80	134 (29.5%)
Level of consciousness	
Conscious/oriented	194 (42.6%)
Disoriented	40 (8.8%)
Light/drowsy sedation	36 (7.9%)
Moderate/torporous sedation	57 (12.5%)
Intense/comatose sedation	268 (58.9%)
Mobility	
No limitation	68 (14.9%)
Little limited	84 (18.5%)
Very limited	35 (7.7%)
Immobility	268 (58.9%)
Friction/shear	
Absent	56 (12.3%)
Present	399 (87.7%)
Comorbidity	
Absent	3 (0.7%)
Present	399 (87.7%)
Turgor/elasticity	
Preserved	3 (0.7%)
Decreased	452 (99.3%)
Tactile sensitivity	
Preserved	186 (40.9%)
Decreased	70 (15.4%)
Absent	199 (43.7%)
Texture	
Hydrated	7 (1.5%)
Oily	1 (0.2%)
Mixed	84 (18.5%)
Dry	363 (79.8%)
Pruritus	
Absent	441 (96.9%)
Present	14 (3.1%)
Pre-existing alterations	
Absent	5 (1.1%)
Photodermatoses	82 (18%)
Deep wrinkles	169 (37.1%)
Flaking	60 (13.2%)
Cracking	139 (30.5%)
Product for hydrating and/or lubricating the skin	
Uses as indicated	90 (19.8%)
Use any available hydrating product	206 (45.35%)
Uses nothing	159 (34.9%)
Hydration routine	
Twice a day	61 (13.4%)
Once a day	232 (51%)
Hydrates without an established routine	10 (2.2%)
None	152 (33.4%)
Water temperature for bathing	
Less than 34ºC	301 (66.2%)
Between 34ºC and 36ºC	9 (2%)
Greater than 36ºC	145 (31.9%)
Body mass index (BMI)	
Between 18.5 and 24.9	182 (40%)
Between 25 and 29.9	167 (36.7%)
Between 30 and 34.9	54 (11.9%)
Between 35 and 39.9	18 (4%)
<18.5	23 (5%)
>40	11 (2.5%)
Phototype	
Black	54 (11.9%)
Dark brunette	56 (12.3%)
Moderate brunette	94 (20.7%)
Light brunette	99 (21.8%)
White	142 (31.2%)
Extremely white	10 (2.2%)
Risk	
Low	17 (3.7%)
Medium	337 (74.1%)
High	101 (22.2%)

Based on the results of the Spearman’s correlation test displayed in Table 4, the variables “level of consciousness”, “mobility”, “tactile sensitivity”, “temperature” and “phototype” showed a positive and moderate correlation in relation to high risk, while the variables “pre-existing alterations”, “age”, “friction/shear”, “BMI” and “texture” showed a positive and low correlation. The variables “turgor/elasticity” and “comorbidity” showed a positive correlation, although it was very low. 

**Table 4:** Spearman’s correlation test and p-value between the ERLAX-53 variables and high risk. João Pessoa, PB, Brazil 2023

Variable	**Spearman’s *r***
Level of consciousness	0.50*
Mobility	0.50*
Tactile sensitivity	0.43*
Temperature	0.41*
Phototype	0.40*
Pre-existing alterations	0.36*
Age	0.31*
Friction/shear	0.31*
BMI	0.25*
Texture	0.24*
Turgor/elasticity	0.17*
Comorbidity	0.08
Pruritus	-0.07
Hydration routine	-0.02
Product for hydrating and/or lubricating the skin	-0.001

* *p* < 0.001

## Discussion

The growing number of hospitalizations of elderly people is proportional to the aging of the population. In view of this increase. Researchers have warned about the control and risk factors for the appearance of skin lesions, and strongly recommend that nurses and their teams be trained in the use of appropriate technologies, as well as emphasizing the need to maintain barrier function, reduce friction and shear, and hygiene care. Despite the wide range of skin care products and brands on offer, some aspects need to be considered, such as purchasing power, choice of product, purpose, skin type and care routine. Regarding this last aspect, although in this study the number of males and females was almost the same, and did not represent significance during the test. In a study conducted in the United Kingdom with two groups of men, one aged between 18 and 27 and the other between 28 and 59, the growing interest in skin care products by men was observed; however, it revealed that this behavior is aimed at improving aspects related to appearance, and also that this routine is something that men use discreetly and usually hide for fear of suggesting feminizing or narcissistic behavior.^12^

This study found that the majority of elderly people lived with family members were retired and had an income of two minimum wages or more. There is a need to pay attention to socio-economic issues, since the lack of products to protect or repair the skin has become commonplace, especially in public or philanthropic hospital institutions, making it necessary for the patient or family member to buy them themselves. When asked why they did not use a product to moisturize their dry skin, they routinely replied that they could not afford it. In addition to this problem, in a study carried out in 2020 that looked at projected spending in Brazil, considering three components: income, demographics and household (that which is not related to demographics or income), it was observed that 26.8% of the increase in the need for health funding was due to the aging population.^13^

When looking at the correlation between the variables on the scale and high risk, level of consciousness and mobility showed a weak, positive correlation and statistical significance. Reducing the level of consciousness in the hospital environment can be a condition of the elderly person or a necessity. Both delirium and sedation compromise autonomy, as well as the response to external injuries and restrict mobility. In a study with a sample made up of elderly people with an average age of 85.4 years, it was identified that xerosis cutis, incontinence-associated dermatitis, other skin lesions, pressure sores (PS) and intertrigo were not associated with each other, however, adverse skin conditions appeared mainly in the long-lived and care-dependent, who had limited mobility.^6^ Regarding tactile sensitivity, a study carried out in England obtained significant results when analyzing the correlation of three variables with age in relation to tactile perception. A negative correlation was found between hydration and age (r=-0.59) and biological elasticity and age (r=-0.46). Nonetheless, there was a positive correlation between the area of perception of the digital pulp and age (r=0.53)^14^. Reduced or absent tactile sensitivity may be a consequence or cause of the appearance of lesions, as in the case of xerosis, a situation which is usually aggravated by a decrease in vascularization, as well as a decline in the production of sweat and sebaceous glands, which also lead to disturbances in thermoregulation.^15^

Temperature in the elderly should be observed and assessed with caution. In a systematic review conducted in Japan, it was observed that among adults and the elderly, the subjective aspects related to the feeling of comfort are, in fact, the items considered. The same study highlights a study that involved bathing infarcted patients in bed at a temperature of 40°C and 42.5°C, and revealed that with the higher temperature, there was an increase in SpO_2_ and a reduction of 1% in heart rate. In this study, the water temperature for bathing in the ICU remained at around 40°C, which is considered high even though it is recommended that the temperature of the environment, through air conditioning, should be between 20 and 24°C, in order to reduce metabolic work, contribute to body temperature control, hemodynamic stabilization, neurological protection and inflammation control.^16^ Thus, the main objective of heating the water for the bed bath routine is to bring comfort to patients; however, in countries with tropical climates, a range between 34° and 36°C has been recommended, considering that hot water removes natural oiliness and further promotes dryness.^17^

It is believed that this and other findings inherent to aspects, such as mobility, alterations in the level of consciousness and friction and shear have been relevant to the positive correlation between the hospitalization sector and the high risk of injury associated with xerosis cutis in hospitalized elderly people. These are environments that offer medium or highly complex care experience bed restriction, the need for frequent decubitus changes, the constant need for assessment and care in the presence of changes in the level of consciousness, as well as injuries caused by friction and shear, which demand material and human resources from nursing, in order to guarantee effective and quality care. 

Another relevant aspect is the skin phototype, since the various methods for characterizing skin type direct care because they guide the selection of the most appropriate products and optimize clinical results. It is imperative to consider that photoaging involves the appearance of wrinkles, spots, dryness and loss of elasticity, aspects that characterize xerosis. There are two phenotypes of photoaging: hypertrophic photoaging, with thick and deep wrinkles, and atrophic photoaging, with a smooth and relatively wrinkle-free appearance, telangiectasia and the presence of lesions.^18^ Photosensitivity can result in various symptoms, diseases or conditions (photodermatoses) caused or exacerbated by exposure to sunlight and ultraviolet radiation, where chronic evolution is characterized by phototoxicity and photoarthralgia. Both conditions, especially in white skin, worsen the process of skin flaking. When associated with the presence of deep wrinkles, irritation and pruritus can lead to the occurrence of cracking.^19^ The sample showed a percentage of 31.2% of people with white skin. Furthermore, when the correlation analysis was carried out with high risk, it was weak, but positive and with a level of significance. 

Another relevant aspect is skin phototype, since the various methods for characterizing skin type guide care, as they guide the selection of more appropriate products and optimize clinical results. It is imperative to consider that photoaging involves the appearance of wrinkles, blemishes, dryness and loss of elasticity, aspects that characterize xerosis. There are two phenotypes of photoaging: hypertrophic photoaging with thick and deep wrinkles, and atrophic photoaging, with a smooth appearance and relatively no wrinkles: telangiectasia and presence of lesions.^18^ Photosensitivity can result in several symptoms, diseases or conditions (photodermatoses) caused or exacerbated by exposure to sunlight and ultraviolet radiation, where the chronic evolution is characterized by phototoxicity and photoarlegia. Both conditions, especially in white skin, worsen the skin flaking process and, when associated with the presence of deep wrinkles, irritation and pruritus, can lead to the occurrence of cracks.^19^ The sample showed a percentage of 31.2% of people with white skin; and when performing the correlation analysis with high risk, it was weak, but positive and with a level of significance.

Friction and shear was a condition present in 87.7% of the sample and one of the extrinsic risk factors, especially for the PS, British researchers underline the importance of knowledge and safety when prescribing or indicating products. When describing a person’s skin receiving a significant shear load through frictional interactions, the result is damage to the stratum corneum, with consequent redness, inflammation, bullous eruptions, open and pressure lesions. It is well known that shear and friction pose a risk to the skin health of elderly people due to reduced blood flow, weakness and cell death, as well as tissue fragility.^20^ A study conducted in Australia, which sought to analyze the prevalence and characteristics of skin lesions in a tertiary hospital over ten years, warned of the importance of training staff, as well as optimizing strategies for the proper handling and transfer techniques of patients, especially with regard to protecting the limbs of older people whose fragile and thinner skin is easily damaged by shear and friction forces.^21^

Although the BMI variables, texture and turgor and elasticity show a negligible level of correlation, their importance should not be invalidated when considering the need for preventive measures. The impact of an inadequate diet goes beyond the prevention and healing of wounds, with subsequent effects that contribute to higher rates of comorbidity. The poor quality of the diet of obese people contributes to inefficient glucose control, dyslipidemia and a worsening of cardiovascular diseases, while malnourished people have a reduced response to health problems, poor circulation and greater susceptibility to infections. A multicenter study showed that nutritional supplements for the prevention and treatment of wounds were associated with a more than four-fold increase in the chances of healing, but it is clear that research on the topic is still incipient.^22^

Nurses routinely inspect the skin on a daily basis and must be vigilant when caring for the elderly patient. Characterizing the texture of the skin will guide the indication and use of products, especially when faced with sensitive skin. A Chinese study testing skin assessment instruments found that the skin barrier was damaged, the texture was rough and there was an active inflammatory process in people with sensitive skin. The importance of assessment and assertive care, using anti-inflammatories and moisturizers (hydrating products), improved texture and hemoglobin, increased hydration of the stratum corneum, reduced transepidermal water loss and decreased the area of sensitive skin.^23^Turgor and elasticity are altered with the progression of aging. Among the alterations, once can mention: thinning of the dermal thickness, changes in collagen and elastic fibers, as well as alterations in the fundamental substance. There is a reduction in the synthesis of type I collagen and a predominance of type III in the elderly patient, as well as an increase in collagenase levels. The amount of mucopolysaccharides decreases in line with the elastic fibers, which become smaller in number and diameter. The decrease in hyaluronic acid also negatively influences the turgor and elasticity of the skin,^24^ which is why its identification alone is indicative of preventive care. 

The lack of correlation in relation to pruritus stands out, as it differs from other studies of elderly people that indicate a prevalence of 21% worldwide and 40% in America. Senile pruritus is defined as generalized pruritus in patients without primary skin lesions, considered to be a common skin disease in the geriatric population that brings with it an unpleasant skin sensation that causes itching and can be accompanied by skin lesions, pain and infection when not treated properly. Risk factors include smoking, excessive alcohol consumption and monophagism.^25^

Conversely, the variable related to “product for hydrating and/or lubricating the skin”, despite being two fundamental aspects for both the prevention and treatment of xerosis, showed no correlation when the analysis was carried out between the two and high risk, probably due to the inherent characteristics of the sample. Moreover, 45.35% use any available moisturizer and 51% apply it at least once a day, which improves the scores on the scale. The main function of a moisturizer is to control moisture loss; however, when the barrier is compromised, it is necessary to first suppress water loss and then restore the barrier. When the moisturizer works properly on the skin, homeostasis is maintained and the oily component creates a film that allows the barrier to be repaired. Therefore, it is agreed that excellent moisturizing agents serve as adjuvants to treat and prevent diseases and also to relieve the symptoms of skin diseases, such as itching and burning.^26^ They are classified as emollients that fill gaps in the stratum corneum, humectants that are substances applied to the surface of the skin, which remove moisture from the atmosphere and moisture below the stratum corneum towards the stratum corneum and, finally, occlusives, which are oily substances that overlay the stratum corneum, as well as having the ability to reduce transepidermal water loss.^27^

Regarding the frequency of hydration, there is still no consensus in the pertinent literature, given the specificities of each skin type, body region and sun exposure, among others. However, clinical trials to test new products routinely do so with twice-daily application.^28^ An Australian study with 443 elderly people over 70 years of age concluded that there were no statistically significant differences for the prevention of skin tears between the intervention and control groups, thus strengthening the need for further research on the topic.^29^

One limitation of the study was the fact that it was carried out in only one hospital, which may compromise the potential for generalization. There is a need to apply the scale in other contexts and profiles of care for the elderly population, since this conduct would enable a more reliable and expanded diagnosis. A collection bias is possible, considering that only two nurses performed it. Finally, a lack of productions on xerosis cutis in Brazil, which compromises the discussion of the research, when considering cultural and economic issues in other countries. 

It is believed that the results found in this study can contribute to the practice and care of nurses in the management of preventive and treatment strategies in the presence of xerosis. It is hoped that the scale can be incorporated into the routine of services, in order to offer support for decision-making and through care, prevent complications and always improve the quality of care for hospitalized elderly people. Further studies should be carried out in other regions, with other patients and other institutions, with a view to observing the characteristics of the patients and whether the results maintain the same profile or not, through the application of scale. 
